# Automatic Bayesian Weighting for SAXS Data

**DOI:** 10.3389/fmolb.2021.671011

**Published:** 2021-06-04

**Authors:** Yannick G. Spill, Yasaman Karami, Pierre Maisonneuve, Nicolas Wolff, Michael Nilges

**Affiliations:** ^1^ Department of Structural Biology and Chemistry, Structural Bioinformatics Unit, CNRS UMR 3528, Institute Pasteur, Paris, France; ^2^ Université Pierre et Marie Curie, Cellule Pasteur UPMC, Paris, France; ^3^ Department of Structural Biology and Chemistry, NMR of Biomolecules Unit, CNRS UMR 3528, Institute Pasteur, Paris, France; ^4^ Center for Molecular, Cell and Systems Biology, Lunenfeld-Tanenbaum Research Institute, Sinai Health System, Toronto, ON, Canada; ^5^ Department of Neuroscience, Current Address, Channel-Receptors Unit, CNRS UMR 3571, Institut Pasteur, Paris, France

**Keywords:** SAXS, bayesian scoring, automatic weighting, inferential structure determination, PTPN4, allosteric regulation, conformational dynamics

## Abstract

Small-angle X-ray scattering (SAXS) experiments are important in structural biology because they are solution methods, and do not require crystallization of protein complexes. Structure determination from SAXS data, however, poses some difficulties. Computation of a SAXS profile from a protein model is expensive in CPU time. Hence, rather than directly refining against the data, most computational methods generate a large number of conformers and then filter the structures based on how well they satisfy the SAXS data. To address this issue in an efficient manner, we propose here a Bayesian model for SAXS data and use it to directly drive a Monte Carlo simulation. We show that the automatic weighting of SAXS data is the key to finding optimal structures efficiently. Another key problem with obtaining structures from SAXS data is that proteins are often flexible and the data represents an average over a structural ensemble. To address this issue, we first characterize the stability of the best model with extensive molecular dynamics simulations. We analyse the resulting trajectories further to characterize a dynamic structural ensemble satisfying the SAXS data. The combination of methods is applied to a tandem of domains from the protein PTPN4, which are connected by an unstructured linker. We show that the SAXS data contain information that supports and extends other experimental findings. We also show that the conformation obtained by the Bayesian analysis is stable, but that a minor conformation is present. We propose a mechanism in which the linker may maintain PTPN4 in an inhibited enzymatic state.

## 1 Introduction

Integrative structural biology uses multiple techniques to determine three-dimensional structures of large, potentially flexible complexes of biological macromolecules. Typically, structures of the individual components (e.g., individual domains or proteins) are known but the overall arrangement of the components is to be determined. Despite their relatively low information content, Small Angle Scattering [Small Angle X-ray Scattering (SAXS), or Small Angle Neutron Scattering (SANS)] experiments play an important role, since they are performed in solution, and can provide crucial conformational information on the arrangement of individual components.

In order to incorporate SAXS data, many approaches generate poses of the components and then use the experimental data to filter solutions by means of a $\chi^{2}$ criterion [e.g., Mareuil et al. (2007); Yang et al. (2010); Rozycki et al. (2011)]. For a larger number of degrees of freedom, or when a large conformational space needs to be covered, this becomes computationally intensive, and one might miss structures that satisfy the data. Preferentially, one would like to employ methods that can use the data directly as restraints to drive the structure calculation, since they should converge faster to conformations satisfying the data. In methods that refine directly against the data, definite choices on unmeasurable model parameters must be made before the minimization. Examples for such parameters are the scale factor between the experimental and the back-calculated data, and the quality or consistency of the data, which has a relationship to the weight on the data employed during the calculation (data with lower quality should get a lower weight). Yet, the optimal weight one should put on the data is never known beforehand. These parameter choices have important consequences, and even more so if SAXS data are to be used together with other data, for which similar problems exist.

When modeling structures from experimental data, appropriate relative weighting is of particular importance. In crystallography, for example, the free R-value Brünger (1992) is often used to find suitable values for unknown parameters such as the weight on the experimental data. This becomes rapidly cumbersome if more than one value needs to be optimized, and it is hardly an option for data with low information content such as SAXS or SANS.

A more powerful and statistically more accurate solution to this problem can be obtained in the context of a Bayesian treatment of the structure determination problem. We previously developed the Bayesian framework we called “Inferential Structure Determination” (ISD) and applied it to Nuclear Magnetic Resonance (NMR) data Rieping et al. (2005). We showed that the Bayesian formalism converges better than standard minimization strategies Rieping et al. (2005). We also showed that an optimal weight on a $\chi^{2}$ type experimental term can be obtained from a 3D structure and the data Habeck et al. (2006), and that this weight can be optimized simultaneously with the structure Nilges et al. (2008), Bernard et al. (2011). More recently, we extended the concept of ISD and Bayesian weight optimization to the treatment of cross-linking mass spectrometry data Ferber et al. (2016) and electron microscopy Bonomi et al. (2019).

In this paper, we develop a Bayesian framework for the analysis of SAXS data. This model allows us to automatically weight the SAXS data based on its agreement with other structural modeling terms. The modeling is performed in several stages, adding additional detail at each stage, starting with rigid body motions of protein domains, and subsequently adding and sampling conformations of the linker and the termini. This is followed by extensive unbiased molecular dynamics (MD) simulation starting from the optimal structure. We apply the new formalism and modelling strategy to the determination of the structure of the tandem domain of the protein PTPN4. This is a good test case since, due to its flexible linker, several conformations may be simultaneously present and influence the measured SAXS data, which hampered previous attempts to obtain useful insights with more standard approaches to interpret SAXS data obtained for this protein.

The protein PTPN4 belongs to the non-receptor protein tyrosine phosphatase (PTP) family. It is involved in various biological processes such as T-cell signalling, learning, spatial memory and cerebellar synaptic plasticity Kina et al. (2007), Kohda et al. (2013), Young et al. (2008). PTPN4 also regulates cell proliferation and presents an anti-apoptotic function Gu et al. (1996), Préhaud et al. (2010), Zhou et al. (2013), Zhang et al. (2019). PTPN4 is a large modular protein containing a N-terminal FERM (Band 4.1, Ezrin, radixin, and Moesin) domain, a PDZ (PSD-95/Dlg/ZO-1) domain and a C-terminal catalytic tyrosine phosphatase domain. The phosphatase is cleaved in the cell, leading to enzyme activation and its active form consists of the PDZ and PTP domains connected by a linker Gu and Majerus (1996). We previously demonstrated that the catalytic activity of the PTP domain is inhibited by the PDZ domain, while the binding of a ligand to the PDZ releases this auto-inhibition and activates the phosphatase Maisonneuve et al. (2014). A biochemical study suggests that this mechanism of regulation of PTPN4 allows for the specific dephosphorylation of cellular partners such as the mitogen-activated protein kinase (MAPK) *p*38*γ* recruited through the PDZ domain of the phosphatase Maisonneuve et al. (2016). The importance of the PDZ domain for PTPN4 is further supported by the fact that the G protein of an attenuated rabies virus strain target this domain to deregulates PTPN4 phosphatase function and ultimately causes neuronal cell death Préhaud et al. (2010), Babault et al. (2011), Caillet-Saguy et al. (2015).

However, the structural mechanism by which the PDZ domain modulates the activity of the phosphatase domain remains elusive. We showed that a conserved hydrophobic patch in the linker connecting the PDZ and the PTP domains is involved in the communication between the two domains and participates in the phosphatase’s regulation Caillet-Saguy et al. (2017). NMR and SAXS characterization of the PDZ-PTP domains of PTPN4 showed that the tandem adopts a compact conformation compatible with inter-domain interactions. However, no interaction was detected by NMR between the phosphatase domain and either the PDZ domain or the unstructured and flexible linker Maisonneuve et al. (2014). This suggests that the compact conformation of the PDZ-PTP domains is stabilized by fuzzy intramolecular interactions. Interestingly, ligand binding to the PDZ domain disrupts the transient interactions of the PDZ domain and the linker with the phosphatase domain. Ligand binding to the PDZ induces dynamic rearrangements of the two domains, resulting in the activation of the phosphatase domain Maisonneuve et al. (2014).

The Bayesian SAXS treatment generates a model of the conformations adopted by the PDZ, linker and phosphatase of PTPN4. This model allows us to propose a mechanism by which the linker can regulate the PTPN4 activity. The structure we obtain is based on the implicit assumption that an ensemble covering a small volume of conformational space can explain the SAXS data. We therefore used the MD simulations to investigate the conformational dynamics of PTPN4 and showed that the proposed preferential relative orientation of the two domains and the linker is stable and corresponds best to the SAXS data. However, the simulations sample other orientations of two domains and the linker, albeit with a worse fit to the SAXS data. By using machine learning and a genetic algorithm we test combinations of structures from the MD trajectories and obtain a dynamic model of PTPN4 that optimally fits the SAXS data.

## 2 Results

### 2.1 Bayesian Small Angle X-ray Scattering Restraint Term

In Bayesian modeling Rieping et al. (2005), one directly evaluates Bayes’ equation

p(X,σ,ξ|B,D)∝p(X|B)p(σ)p(ξ)p(D|X,σ,ξ) (1)

where *X* is the 3D structure, *σ* is a parameter quantifying the deviation of the back-calculated data from the experimental data, and *ξ* stands for any other unknown parameters that one needs to model the data from the structure. *B* is the background information that we have on the structure, which allows us to evaluate the probability of a structure in absence of experimental data, for example, a molecular dynamics force field. To evaluate the discrepancy of the calculated data from the experimental data, we need a forward model m(X) to calculate the intensities ℐ=m(X) from a structure *X*. We used the FoXS model Schneidman-Duhovny et al. (2013), which has, in addition to a scale factor *γ*, two parameters $c_{1}$ and $c_{2}$, where $c_{1}$ is the scaling of the atomic radius used to adjust the total excluded volume of the atoms, and $c_{2}$ is used to adjust the difference between the density of the hydration layer and the bulk water.

As derived in detail in the Appendix, the negative log likelihood is

$$-\text{log}p\left(I|X,\gamma ,c_{1},c_{2},\sigma^{2}\right)=\frac{M}{2\sigma^{2}}\chi^{2}+M\text{log}\left(\sigma \right)$$ (2)

$$\chi^{2}\equiv \frac{1}{M}\overset{M}{\underset{i=1}{\sum }}{\left(\frac{I\left(q_{i}\right)-\gamma m\left(X,q_{i},c_{1},c_{2}\right)}{s\left(q_{i}\right)}\right)}^{2}$$ (3)

where I is the experimental intensity, *M* is the number of points in the SAXS profile, $q_{i}$ is the momentum transfer q=(4πsin(θ))/λ, with scattering angle *θ* and X-ray beam wavelength *λ*. $s\left(q_{i}\right)$ is the experimental uncertainty of the SAXS profile at $q_{i}$ estimated from merging multiple experimental profiles.

### 2.2 Application to Protein Tyrosine Phosphatase Non-Receptor 4

To illustrate the Bayesian SAXS score, we perform exhaustive sampling of the conformational space of the PDZ and PTP domains of PTPN4, which for simplicity we call PTPN4. The PDZ (92 residues) and PTP (275 residues) domains are connected by a linker of 34 residues, and flanked by N-terminal (13 residues) and C-terminal (13 residues) sequences. The structures of individual domains are known Babault et al. (2011), Barr et al. (2009). However, the linker and the termini are highly flexible as monitored by NMR Maisonneuve et al. (2014). They thus prevented the determination by X-ray crystallography of the overall organization of the two domains of PTPN4 tethered by the linker.

**FIGURE 1:**
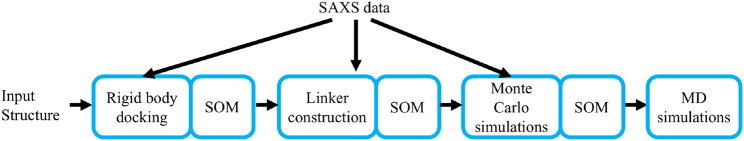
The workflow of the method. The main steps of the algorithm are depicted: rigid body docking, linker construction, Monte Carlo simulations, and Molecular Dynamics (MD) simulations. The Small-angle X-ray scattering (SAXS) data is used to derive the first three steps.

To efficiently characterize the structural conformation of PTPN4 by a Bayesian SAXS score, we subdivided the problem into three subsequent stages (Figure 1 **)**. First, the linker and the termini were removed and the conformational space was explored with rigid body movements of the folded domains. Second, linker and termini were added, while keeping the domains fixed. Third, the whole structure was further refined with rigid body movements for the two domains and flexible backbones for the linker and the termini. In all three stages, we used Eqs. 2,3 to incorporate the SAXS profile of PTPN4. Volume exclusion was used to produce physically realistic structures.

#### 2.2.1 Rigid Body Docking

**FIGURE 2:**
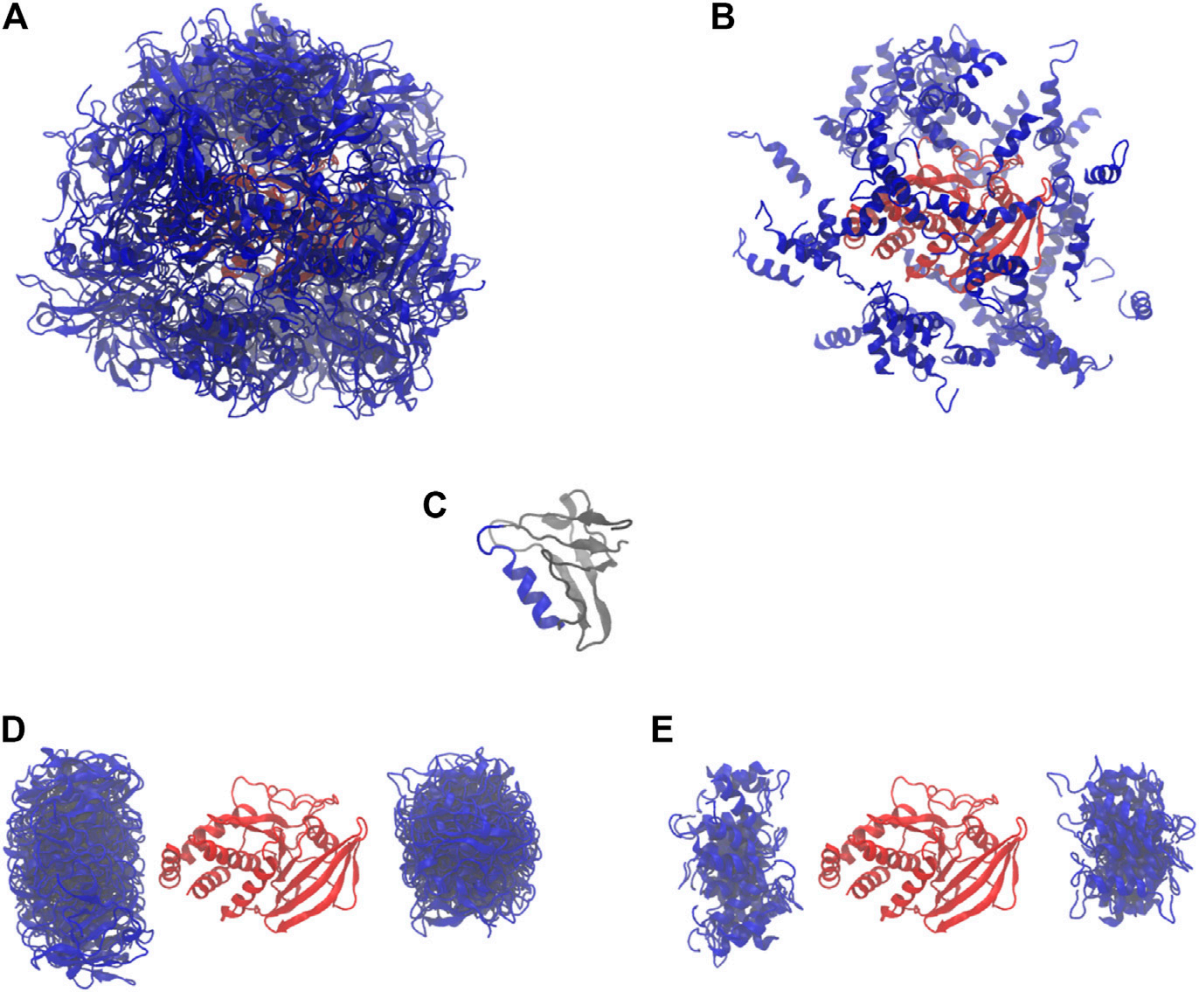
Starting and final conformations of the 64 rigid body simulations. PDZ in blue, PTP in red. **(A)** Starting conformations (full PDZ). **(B)** Starting conformations (only α2-helix for PDZ). **(C)** PDZ, with α2-helix in blue. **(D)** Final conformations (full PDZ). **(E)** Final conformations (only α2-helix for PDZ).

We started with 64 parallel simulations by placing the PDZ domain randomly around the PTP domain (without the linker and termini), avoiding physical contact between the two proteins (Figures 2A,B). The simulations rapidly converged to two distinct sets of conformations in which the PDZ domain (Figure 2C) is located on either of the two most distant points of the phosphatase domain, each subdivided in two further conformations (Figures 2D,E). In these conformations, the *α*2-helix of the PDZ domain is roughly aligned with the main axis of the phosphatase domain. This indicates a preferred orientation of the PDZ domain relative to the PTP domain.

**FIGURE 3:**
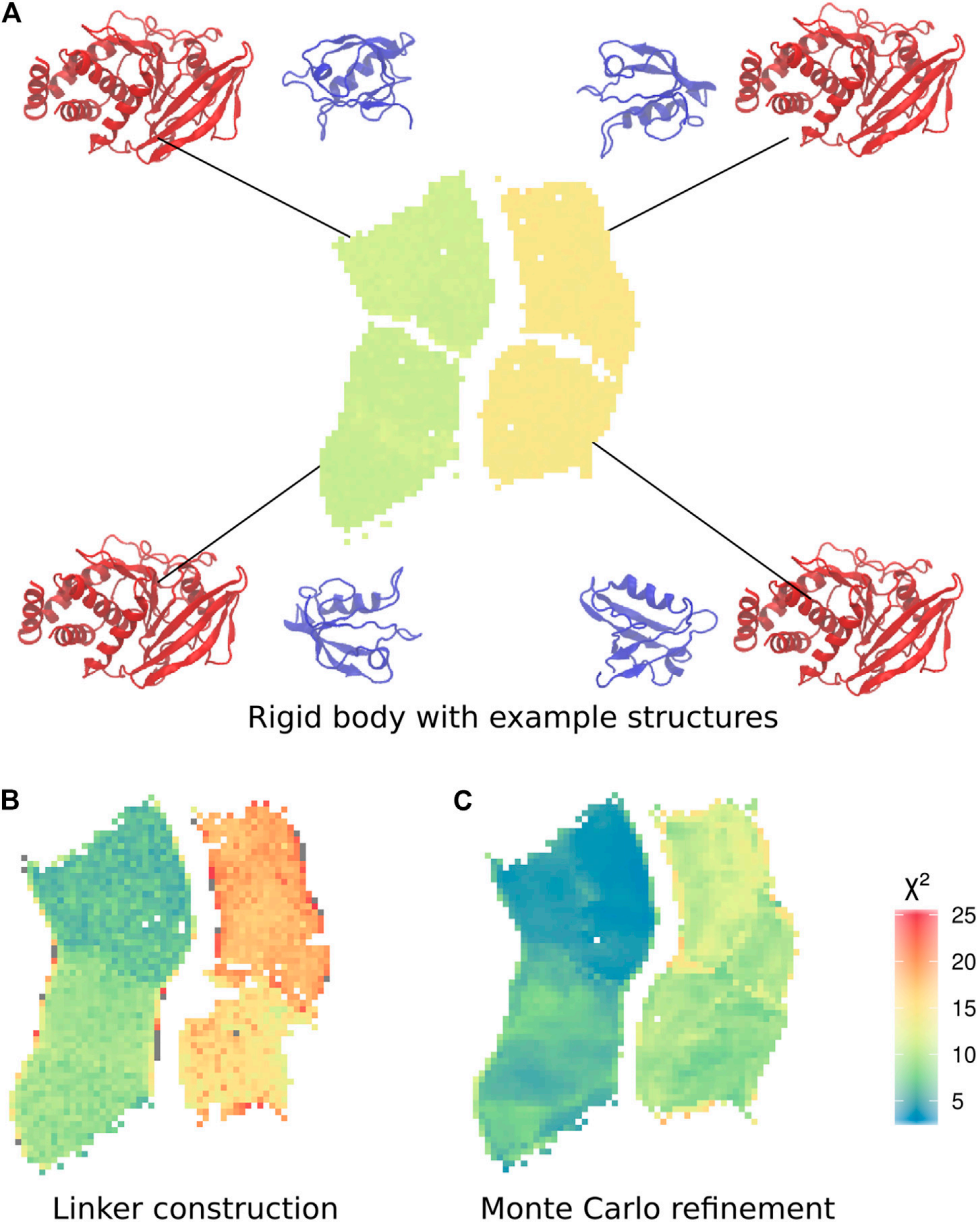
Self-organizing maps (SOMs) of the three calculation stages. **(A)** Final conformations of the rigid body docking stage, coloured by $\chi^{2}$. Only SOM neurons with at least one structure are shown throughout. For each cluster, an example structure shows the relative orientation of the PDZ with respect to the phosphatase domain. **(B)** Best $\chi^{2}$ for each docking pose with linker added. **(C)** Average $\chi^{2}$ of minimized linker conformations.

To analyse the trajectories, we trained a self-organizing map (SOM) Bouvier et al. (2015). The subdivision of the two distinct sets of conformations into two further sets is clearly visible in the SOM, making it possible to define a total of four clusters (Figure 3A). Each cluster corresponds to one of the four possible combinations of position of the PDZ domain, and orientation of the *α*2-helix of the PDZ domain, with respect to the main axis of the phosphatase domain.

#### 2.2.2 Linker Construction

We then extracted a clash-free conformation displaying the lowest $\chi^{2}$ for each of the neurons of the SOM (Figure 3A). For every selected structure, we generated an average of 1,224 conformations for the linker and the termini sequences (see Methods). A Bayesian SAXS score was calculated for each of these structures. Depending on the pose, the linker raised or lowered the Bayesian SAXS score (Figure 3B). For each neuron we retained the structure with linker and termini displaying the lowest $\chi^{2}$ (Figure 3B). Interestingly, the models with the lowest Bayesian SAXS scores are located in the two left clusters of the SOM corresponding to PDZ domains exclusively located on the side of the PTP *β*-sheet (Figure 2E). These clusters differ in a rotation of the PDZ by 180°. In these conformations, the attachment points of the linker to the PTP domain are located on the opposite side from where the PDZ domain is positioned. This implies that the linker passes over the surface of the phosphatase to reach the PDZ domain.

#### 2.2.3 Monte-Carlo Refinement

To further improve the sampling of the conformational space of the linker and termini, we performed an exhaustive refinement of the best structures of each neuron of the SOM map. We used a Monte-Carlo algorithm to sample the linker conformations in the dihedral angles of the linker and termini. As previously, we used only the Bayesian SAXS scoring term and volume exclusion to calculate the energy. This approach allowed the added residues and the domains to adjust jointly to the SAXS profile. The $\chi^{2}$ significantly improved compared to the previous step for all clusters (Figure 3C), and values lowered by 36% on average to a range between 3 and 18. However, the trend in the four clusters remained the same. The structures with the lowest $\chi^{2}$ scores after Monte-Carlo simulations belong to the cluster in the upper left corner of the self-organizing map as previously observed in the step of linker construction (Figure 3C). This indicates that the linker passes over the surface of the phosphatase for the structures which are in best agreement with the SAXS data.

**FIGURE 4:**
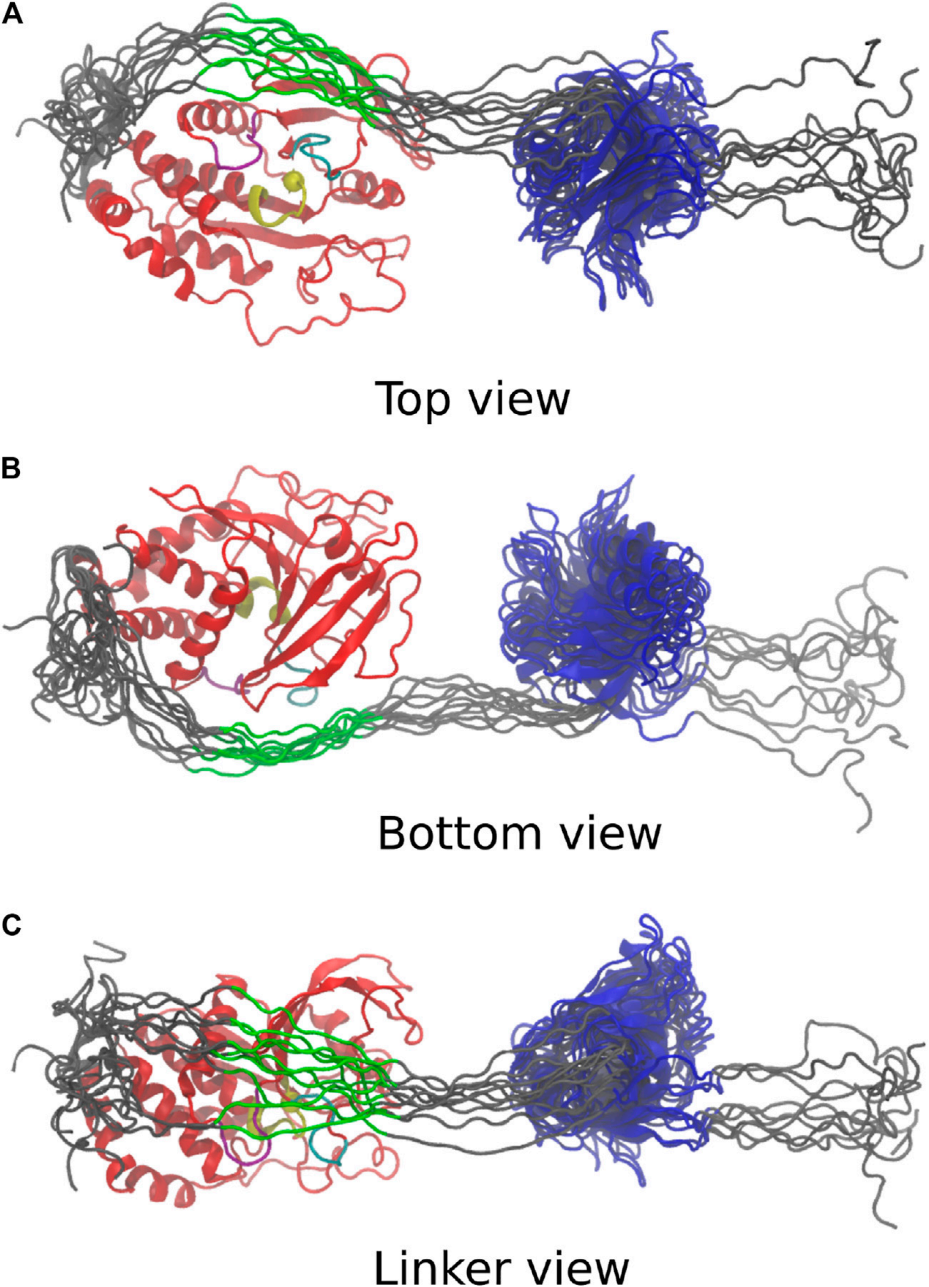
Last frame of the top 10 simulations, aligned on the PTP domain. PTP: red; PTP loop and catalytic cytosine [H (851)CSAGIGRT (859)]: yellow; WPD loop [W (818)PDHGVP(824)]: purple; *β*5-loop-*β*6 region [T (754)QVERGRV (761)]: cyan; C-terminus, N-terminus and linker: grey; highly conserved linker region [E (617)PDFQYIP(624)]: green; PDZ: blue. **(A)** Top view depicts catalytic site in vicinity to the linker. **(B)** Bottom view adopts same orientation as Figure 3. **(C)** Linker view shows conformational variability of linker.

The 10 conformations with the best final $\chi^{2}$ after Monte-Carlo simulations, ranging from 2.5 to 2.9 are presented in Figure 4. In these 10 conformations, the linker is wrapped around the phosphatase domain and passes in close proximity to the catalytic site of the phosphatase domain. Interestingly, a conserved sequence in the linker (shown in green), involved in the allosteric regulation of PTPN4 Caillet-Saguy et al. (2017), is facing both the *β*5-loop-*β*6 region and the WPD loop, a conserved catalytic motif. This observation suggests a possible effect of the linker on these two regions.

#### 2.2.4 Influence of the Weight Adjustment

**FIGURE 5:**
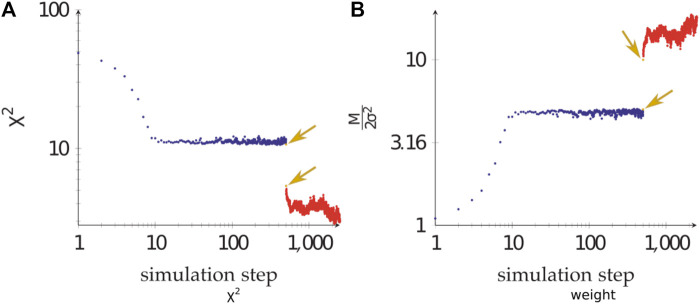
The adjustment of the Bayesian SAXS score. **(A)** $\chi^{2}$ and **(B)** SAXS restraint weight of a composite simulation, starting from rigid body steps (blue), followed by linker modelling (yellow, pointed to by arrows), and ending with Monte Carlo flexible refinement (red).

**FIGURE 6:**
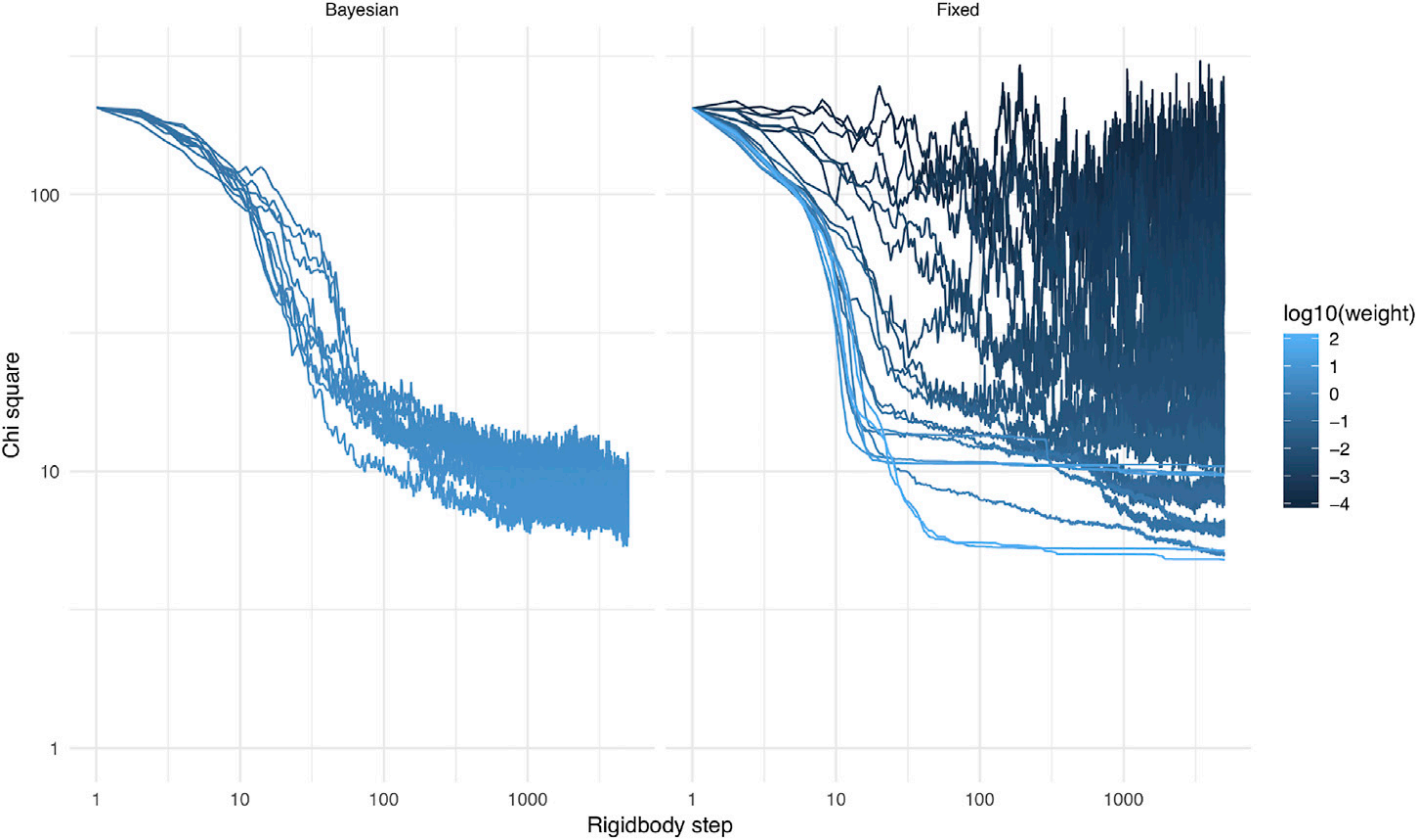
$\chi^{2}$ score as a function of simulation step, for the 64 rigid body simulations with Bayesian SAXS score (left), and for 20 simulations with a fixed weight (different in each simulation) and the same, random, starting structure (right).

During the calculations, the weight of the Bayesian SAXS score adjusted substantially (Figure 5). From the initial rigid body docking to the best structure after refinement, the weight was multiplied by 17. This means that the SAXS data was given 17 times more importance at the end of the procedure compared to the beginning. To see why this matters, we performed 20 linker refinement simulations with a fixed weight for the SAXS restraint, varying from 10^−4^–10^2^ and compared it to 10 simulations using the Bayesian SAXS restraint. We then examined the $\chi^{2}$ along the simulation step, for all replicates (Figure 6). All Bayesian SAXS simulations consistently reach low $\chi^{2}$ values. In contrast, two limiting cases emerge in the fixed-weight simulations. When the weight is very large, agreement to the SAXS data is substantial, and the simulation quickly finds a local SAXS restraint minimum. Sometimes, conformers with can be obtained, but more often less optimal basins are targeted, with $\chi^{2}∼10$ in this example. The Monte Carlo acceptance rate then drops to zero, and the simulation stops exploring new conformations. In contrast, when the weight is very small, the SAXS score has little influence. The simulation can scan conformational space easily, but it has no chance of finding structures in good agreement with the SAXS data.

### 2.3 Stability of the Optimal Conformation

#### 2.3.1 Molecular Dynamics Simulations and Conformational Clustering

**FIGURE 7:**
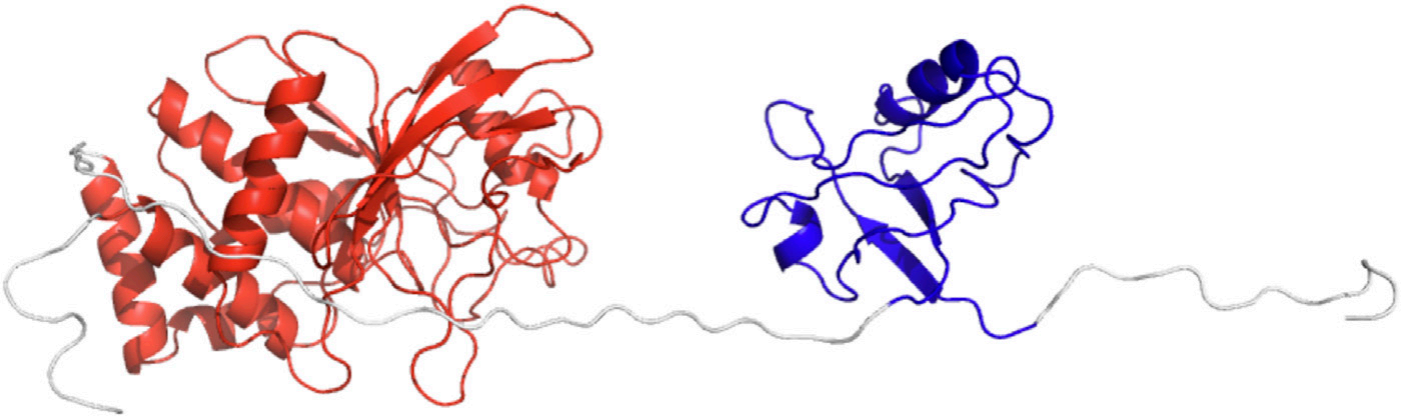
The cartoon representation of starting conformation for the MD simulations. PDZ is colored in blue and PTP in red.

**FIGURE 8:**
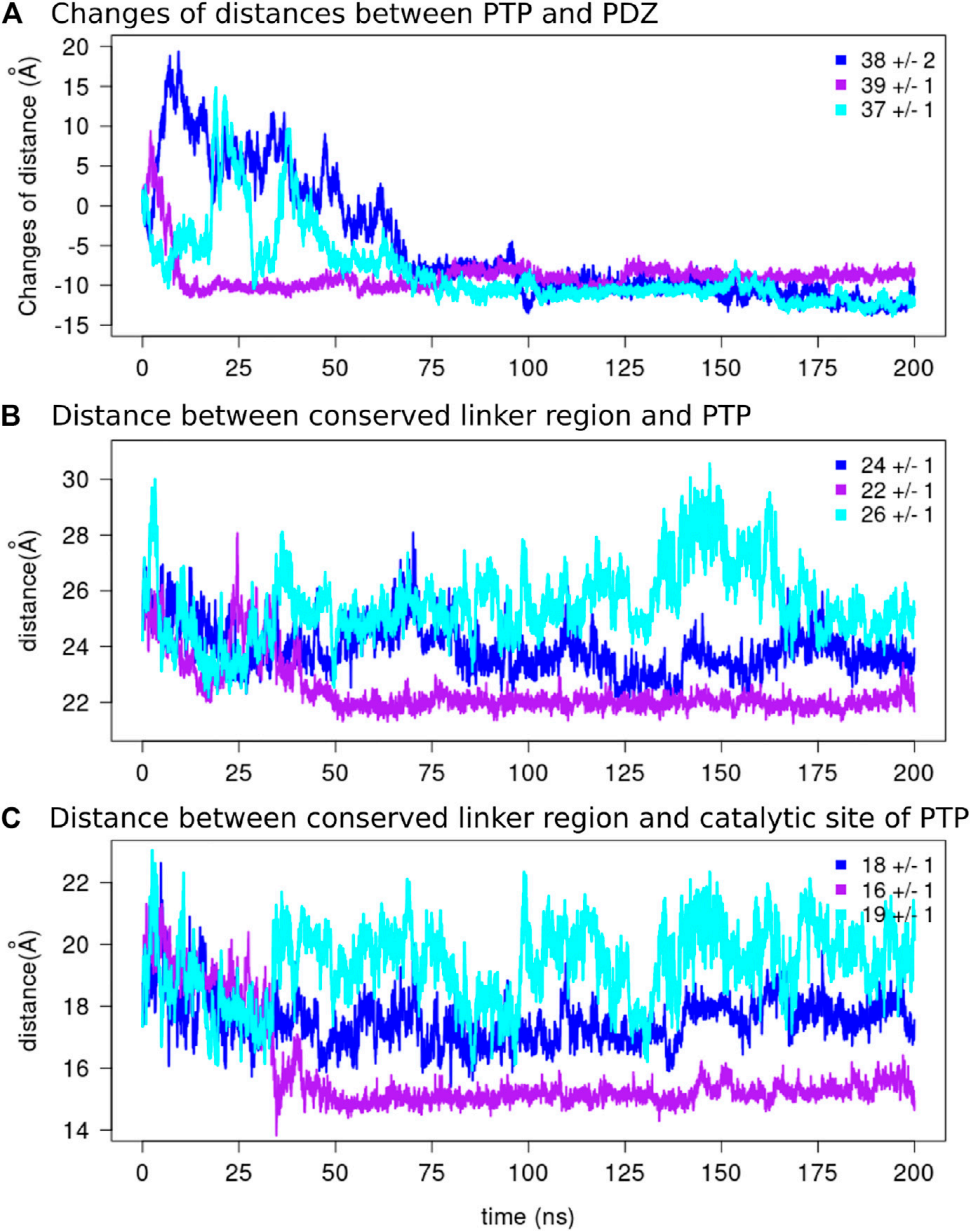
The distances along the MD simulations.**(A)** The changes of distances between the two domains and the average values over the final 125 ns of the simulations are reported for each replicate. The distance between the center of mass of the conserved linker region [E (617)PDFQYIP(624)] and the center of mass of **(B)** the PTP domain and **(C)** the catalytic site of the PTP domain are depicted for each replicate.

To further assess the stability of the optimal conformation obtained from the Bayesian analysis, we performed three MD simulations of 200 ns starting from the model with lowest $\chi^{2}$ = 2.48 (Figure 7). Initially, the relative position of the two domains fluctuates, but it converges in each case to a more compact structure with direct and stable interactions between the two domains after a maximum of 75 ns. This behaviour is reflected in the analysis of the distances between the two domains (Figure 8), showing an initial increase of the distances (∼ 9–18 Å) followed by gradual reduction of distances (∼ −10 Å), with respect to the initial conformation.

**FIGURE 9:**
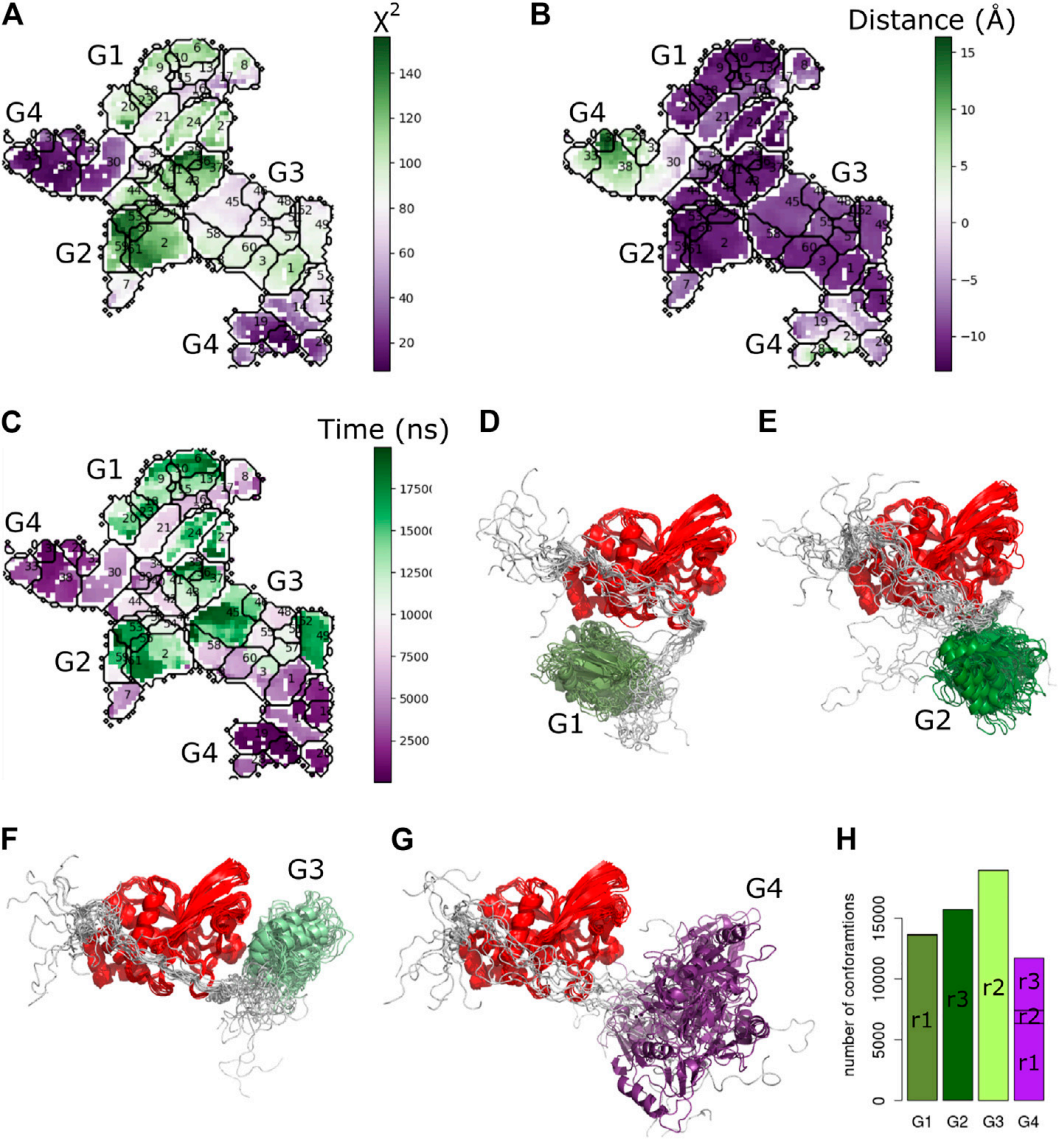
Cluster analysis of PTPN4 from the MD simulations. The self-organizing map of the PTPN4 conformations colored by **(A)** $\chi^{2}$, **(B)** changes of distances between the center of mass of the PDZ and PTP domains, and **(C)** simulations time (ns) of the replicates. The clusters are numbered on the maps from 1 to 60, and divided into four groups (G1-G4). One representative conformation is shown for the clusters that are forming the four groups G1, G2, G3 and G4 in **(D–G)**, respectively. The PTP is colored in red and the four identified groups of PDZ clusters in difference shades of green and purple, reflecting their average $\chi^{2}$ values. **(H)** The number of conformations from different replicates (r1, r2, and r3) are reported for each group.

To better characterize the observed conformational transitions along the MD simulations of PTPN4, we clustered the set of conformations with the Self Organizing Maps (SOM) method already used above Bouvier et al. (2015). A total of 60 clusters were retrieved from a pool of 60,000 conformations (Figure 9). We then projected the $\chi^{2}$ values, the changes of distances between the two domains and the simulation time on the SOM map (Figures 9A–C). The analysis of the two maps suggested four groups of clusters, where G2 had the highest $\chi^{2}$ and maximum increase of distances, and G4 the lowest $\chi^{2}$ and minimum changes of distances. Figures 9D–G shows one representative conformation per cluster, clearly indicating four distinct relative positions of the PDZ with respect to the PTP. These four conformations satisfy the SAXS data to a very different degree, indicated by the color in the SOM maps and in the conformations shown (each group is colored according to their average $\chi^{2}$ value from dark violet for the minimum values to dark green for the maximum values). The analysis of the PTPN4 conformational changes revealed the existence of four distinct conformational states for the PDZ with respect to the PTP, one of which is close to the Bayesian SAXS restraint model and has a low $\chi^{2}$.

To investigate overall convergence of the simulations, we analyzed the number of conformations from different replicates in each group (Figure 9H). The three replicates cover rather different conformational space. The groups G1, G2, and G3 contain conformations from only one replicate. Interestingly, only G4, which is the closest one to the starting conformation and has the lowest $\chi^{2}$ scores, contains conformations from all the three replicates. The further analysis of the clusters along the simulation time (Figure 9C) showed that G4 contains trajectories appearing at the beginning of the simulations, and the G1-G3 are visited subsequently. Interestingly, the position of the linker with respect to the PTP, remained unchanged in all the clusters as can be seen in Figures 9D–G. In order to further investigate the conformational changes of the linker, we measured the distances between the center of mass of the conserved linker region (E617-P624) and i) the PTP domain and ii) the catalytic site of the PTP domain (the *β*5-loop-*β*6 region and the WPD loop) along the three replicates of the MD simulations (Figures 8B,C). The variation of distances are within the range of 1 Å, therefore suggesting the rather stable position of the linker with respect to the PTP domain.

#### 2.3.2 Selection of Minimal Small Angle X-ray Scattering Ensemble

**FIGURE 10:**
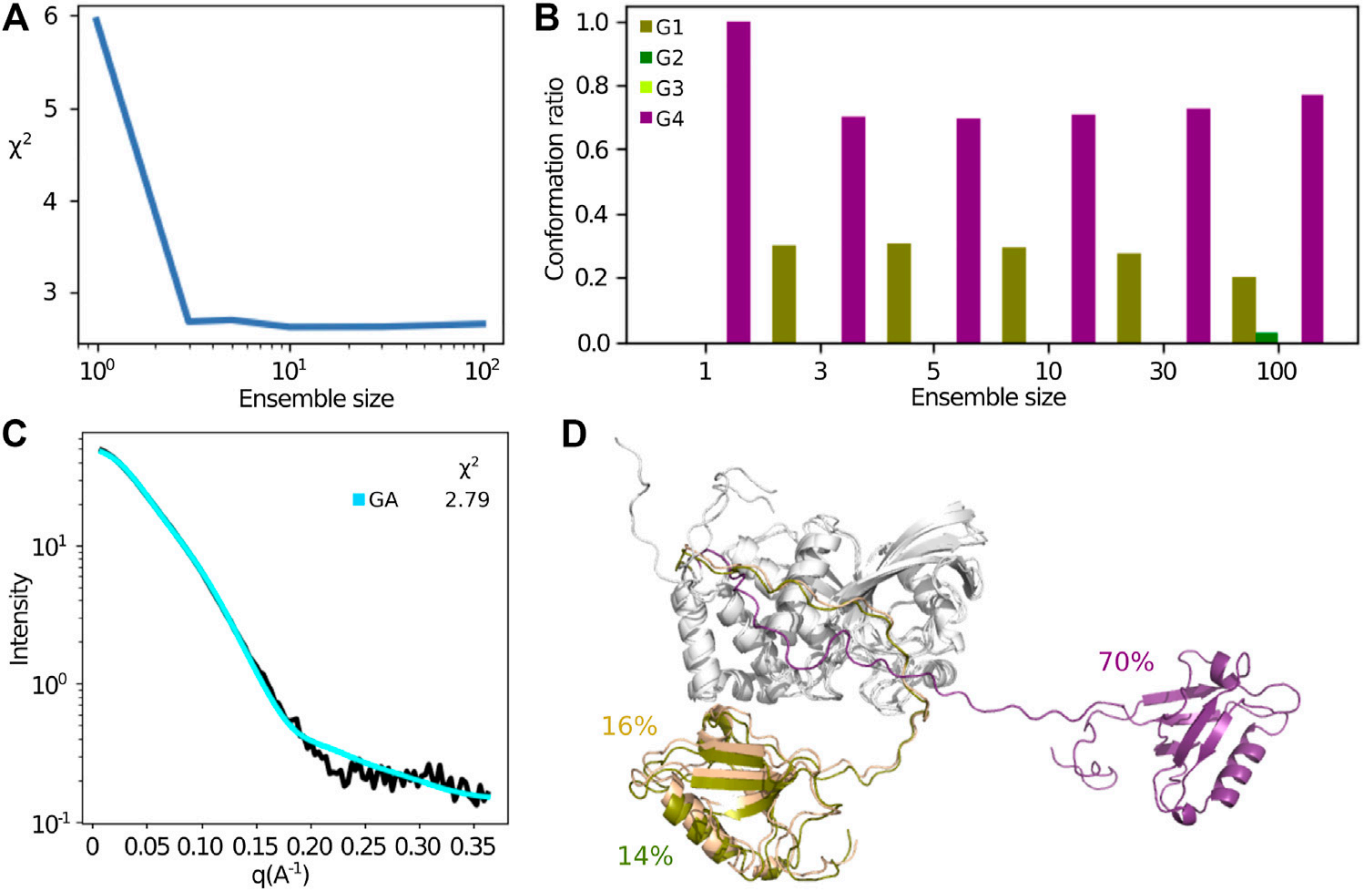
Extracting a minimum subset of conformations from the MD simulations, that describes best the SAXS data using a genetic algorithm. **(A)** Improvement of the $\chi^{2}$ with respect to the ensemble size in the genetic algorithm selection. **(B)** The conformations are clustered in four categories according to the clustering obtained using SOM; conformations in the groups G1, G2, G3, and G4 are colored in olive, green, yellow-green and purple bars, respectively. The proportion of each group is represented for each ensemble size. **(C)** Back-calculated SAXS profile using the genetic algorithm derived from the ensemble size of three (in blue) and the experimental profile (in black), with the $\chi^{2}$ = 2.79. **(D)** Representative ensemble of PTPN4 superimposed on PTP showing the PDZ in G1 conformation (olive and khaki) or in G4 conformation (purple) with the proportion of each conformer.

The above analysis assumes that a single structure or an ensemble covering a small part of conformational space represents the SAXS data. The sampling of conformational space by the free MD trajectories enabled us to try to investigate if more disperse ensembles fit the SAXS data better. For this, we used a method based on the genetic algorithm (GA) that was developed for a similar problem Delhommel et al. (2017). This method searches for the minimal subset of conformations minimizing the error between the experimental data and computed data from the MD simulations. The $\chi^{2}$ values obtained after fitting were reduced from 6.03 to 2.79 for an ensemble size of three. Increasing the ensemble beyond three did not reduce the $\chi^{2}$ further (Figure 10A). We clustered the weighted conformations obtained in all the ensembles according to the four conformational groups identified by the SOM analysis (G1, G2, G3, and G4). Figure 10B shows the ratio of conformations that belong to each group for each ensemble size, averaged over the 5 GA runs. The ratios of the conformations belonging to the four groups are similar for different ensemble sizes, where G4 is always most represented with a weight of about 70%, while G1 has about 30% of the weight. The experimental and fitted profiles (for the ensemble size of three) are compared (Figure 10C, shown in black and cyan, respectively), and the conformations obtained for the ensemble size three are shown in Figure 10D. We conclude that the SAXS data are best represented by two major conformations, an open and a closed states. The open state has the highest weight (70%) and is similar to the initial conformation obtained by the Bayesian method.

## 3 Discussion

### 3.1 Automatic Weight Adjustment

In general, and also in the Bayesian formalism, the SAXS scoring term is based on $\chi^{2}$ (Eq. 3), here multiplied by a weight $M/2\sigma^{2}$. Commonly, the weight on the scoring term is based on some heuristics, for example the number of independent data points Shevchuk and Hub (2017). Experience shows that this weight is not easy to set and can require adjustment during the simulation, in particular when $\chi^{2}$ is expressed with SAXS intensities (as opposed to their logarithms) Chen and Hub (2015). In the context of the Bayesian formalism, the weight is set by changing *σ*. This parameter does not only depend on the quality and consistency of the experimental data but also on the forward model used. The nuisance parameter *σ* evidently scales the experimental errors with a constant factor, and it is unknown before the calculation. It is the hallmark of the Bayesian formalism that this parameter is treated as an unknown, at the same level as the coordinates. *σ*, and in consequence the weight, is adjusted during the calculation, without making any additional assumptions on the values it can take. To do this, we use the second term on the right hand side of Eq. 2, Mlog(σ). In absence of this term proportional to the logarithm of *σ*, the trivial minimum of the score would be reached when *σ* diverges and the weight becomes zero. This automatic weighting modulates the effect of $\chi^{2}$ on the final scoring term. This treatment is analogous to what we introduced for NMR data, electron microscopy data Habeck et al. (2006), Nilges et al. (2008), Bernard et al. (2011) and cross-linking mass spectrometry data Ferber et al. (2016).

### 3.2 Influence of the Weight Adjustment

As an illustration, suppose structure determination is performed with a bad guess for the initial structure. In this case, $\chi^{2}$ will be large. Adjustment of the weight will drive *σ* towards larger values, and the weight becomes smaller. *σ* acts to reset the scale of the restraint. Notice however that its update is less frequent than that of $\chi^{2}$. That way, structures are sampled with $\chi^{2}$ values around $\sigma^{2}$, which is then slowly lowered to increase stringency on the restraint. $\sigma^{2}$ acts as an annealing parameter. As long as the structure is in strong disagreement with SAXS data, the weight of the Bayesian SAXS score will be small. This behaviour allows other terms of the force field to dominate, and conformational exploration can happen unhindered by an irrelevant SAXS term. If exploration leads to a structure with a smaller $\chi^{2}$, the weight will increase. The SAXS term therefore becomes more discriminant, guiding the calculation to propose structures which match the SAXS profile more closely. Bayesian formulation of SAXS structure determination therefore transforms a rugged energy landscape into a funnel-shaped landscape Dill and Chan, 1997.

Note that, the *σ* is being adjusted on the fly, and the maximum likelihood estimate of *σ* is approximately $\chi^{2}$ (Supplementary Equation S4). Therefore, the proper quantity to look at is $M/2\sigma^{2}$ (see Figure. 5B), which is a function of the degrees of freedom in the curve (Spill, 2013) (Section 2.4.8.3, pp 171). In case of multiple datasets, it is therefore crucial that each has their own *σ*.

### 3.3 Fixed Weight vs. Bayesian Automatic Weighting

The optimal weight, at which the simulation has reasonable acceptance rates and makes good use of SAXS information, is *a priori* unknown. It is the purpose of the Bayesian SAXS restraint to determine this optimal weight. As shown in Supplementary Equation S4 (see Supplementary Material), the number of SAXS data points and the overall agreement of data and structures will greatly influence the optimal weight. Therefore, it is expected that it will be different for each SAXS dataset, but also for each simulation setting, for example depending on which force field is used.

### 3.4 Log Score vs. Linear Score

An equivalent form for the Bayesian score without any additional parameter *σ* can be derived by an operation called marginalization (Supplementary Equation S5, Supplementary Material). As shown for NMR data Habeck et al. (2006), this form is equivalent to the weighted $\chi^{2}$ term, but does not need automatic weight adjustment, because it incorporates the behavior described above. Its form is simply the logarithm of the traditional $\chi^{2}$. Using the logarithm of the $\chi^{2}$ lowers the score penalty for large values of $\chi^{2}$, while keeping its effect similar to the standard $\chi^{2}$ formulation when it is close to one. Interestingly, it has been observed by Chen and Hub (2015) that a $\chi^{2}$ formulation using the logarithm of the intensities does not require much adjustments of the weight. While they apply the logarithm on the individual intensities and not the $\chi^{2}$ as a whole, the effect of lowering the impact of large discrepancies remains. When using a $\chi^{2}$ on linear scale (as proposed here), the authors observe the need to adjust this weight specifically in the beginning of the simulation. That is, when discrepancies in the low-q and high diffusion intensity region of the SAXS curve are likely to occur, and contribute most to the scoring. Applying a logarithm on the first part of the SAXS curve is therefore what probably alleviates the need to adjust the weight. In contrast, we have employed a $\chi^{2}$ on a linear scale (including error bars, Eq. 3) because the SAXS measurements and noise scale linearly. The logarithm is applied afterwards, for scoring purposes.

### 3.5 A Point on Exhaustivity

The calculations presented here attempted to sample a large part of the conformational space of this two-domain system, since the energy landscape can be expected to be rugged. We showed that the energy surface is less rugged when using automatically adapted weights. The strength of this Bayesian restraint is that, regardless of the initial conformation, the calculations converge to low $\chi^{2}$ structures. This is particularly beneficial when computer resources are limited. In our PTPN4 example, one in every four simulations ends up in the basin with the lowest $\chi^{2}$ conformers.

### 3.6 Protein Tyrosine Phosphatase Non-Receptor 4

Using the novel Bayesian SAXS restraint, we have shown a conserved sequence in the linker of PTPN4, involved in the allosteric regulation of PTPN4 Caillet-Saguy et al. (2017), is facing both the *β*5-loop-*β*6 region and the WPD loop. The *β*5-loop-*β*6 region is thought to participate in defining substrate specificity Andersen et al. (2001). The WPD loop is well-known to be important for the phosphatase catalysis. The WPD loop switches from an open to close position upon substrate binding and adopts a catalytically active close conformation Barr et al. (2009). Previous experimental evidence showed that the linker participates in the control of the catalytic activity of the phosphatase domain Maisonneuve et al. (2014).

Mutations of a conserved hydrophobic patch in the linker suggested that the linker modulates the WPD loop open/closed conformations Caillet-Saguy et al. (2017). The close proximity of the linker with the *β*5-loop-*β*6 region and the WPD loop observed in our simulations further supports and reinforces the current model in which the linker of PTPN4 could regulate the phosphatase activity of PTPN4 by modulating the WPD loop closure.

### 3.7 Ensemble Modelling

The focus of this study is to illustrate the power and utility of the Bayesian SAXS score. The setup was deliberately simple, to emphasize to what degree the final conformations were driven by the data. Emphasis was also on calculation efficiency, and the molecule was deliberately described in the simplest terms by excluding volume, rigid bodies for the two domains, and rigid covalent geometry. The experimental data was limited to SAXS data up to q<0.37Å${-}^{1}$. The SAXS data do not contain any information on specific interactions between the linker and the surface of the PTP domain. In our models the linker wraps around the PTP domain but does not directly contact the domain. This is consistent with the fact that there is no experimental NMR data that indicates a specific contact, but does not explain the sequence conservation in the linker and on the surface of the PTP domain. The tandem of PDZ-PTP domains in PTPN4 may be the location of continuous conformational changes due to the fuzzy nature of the intramolecular interactions that stabilize the spatial organization of the two domains Maisonneuve et al. (2014, 2016), Caillet-Saguy et al. (2017). This is further confirmed by the analysis of conformations generated by MD simulations starting from the top model, where four distinct groups of conformations are identified (Figure 9). The flexible and unstructured linker is most likely in transient interactions with the PTP domain as monitored by NMR (R2 relaxation rate, Maisonneuve et al. (2014) Figure 5B). In our calculations, the models with low-$\chi^{2}$ (the upper left cluster in Figure 3C) present conformations of the linker that covers almost half of the PTP domain. This conformation of the linker with respect to the PTP domain remains rather stable along the MD simulations.

The conformations we obtain can serve as the basis of more detailed simulations with state of the art ensemble methods Potrzebowski et al. (2018), Shrestha et al. (2019), Paissoni et al. (2020). For a system of rather moderate size as the PTPN4 tandem (52 kDa), one could obviously directly refine against the data in a complete force field Shevchuk and Hub (2017). This would not allow for as extensive searching of conformational space as it was performed in this work. The aim of the current calculation protocol is to sample large relevant parts of conformational space efficiently, a task that is difficult to perform for large fully solvated molecules. An adaptation of the Bayesian SAXS restraint with automated weighting as described here could be useful also in this context. We note that such adaptation however, would not address the issue of multiple conformations representing the SAXS data. In this study we proposed a method to overcome this problem by first concentrating on obtaining the dominate conformational ensemble in a largely simplified force field without explicit solvent, and then further exploring a larger ensemble by a free, fully solvated simulation, and finally obtaining an optimal, small ensemble by combining different conformations from these simulations. While the best conformer obtained by Bayesian SAXS restraint has $\chi^{2}$ = 2.48, our approach allowed us to reveal an ensemble of three structures capturing two different states of PTPN4 with a fitted $\chi^{2}$ of 2.79. Interestingly, while for several of the proteins studied with the CHARMM36m force field, the resulting structures are more compact than indicated by experiment (unless protein-water interactions are increased) Huang et al. (2017), our analysis highlights both compact and open states for PTPN4.

## 4 Materials and Methods

### 4.1 Protein Production and Data Collection

The PDZ-PTP^C/S^ construct, harboring the mutant C852S, hereafter referred to as PTPN4, was expressed and purified as previously described Maisonneuve et al. (2014). SAXS experiments were carried out as previously described except that the protein concentration used for SAXS experiments was 75 μM Maisonneuve et al. (2014).

### 4.2 Rigid Body Docking

In the first stage, we used IMP Russel et al. (2012) to perform rigid body docking of the PDB structures of PTP (PDB code 2I75; residues 638–913) and PDZ (PDB code 3NFK chain B; residues 512–604). 64 different simulations were performed with 500 steps each. Initial orientations of PDZ with respect to PTP cover a wide range of orientations both around the PTP and of the PDZ itself (see Figure 2). Energy terms were the SAXS restraint (Supplementary Equation S7) and a quadratic volume exclusion term. The FoXS model was used on heavy atoms Schneidman-Duhovny et al. (2013). Each step consisted in alternating 100 Monte Carlo rotation/translation moves $(510^{-2}\text{rad}/$Å) of PDZ with respect to PTP, and optimizing $c_{1}$, $c_{2}$, *σ* and *γ*. *σ* and *γ* were optimized by setting them to their maximum posterior (Supplementary Equation S4 and Spill et al. (2014)). $c_{1}$ is constrained to be between 0.95 and 1.05, while $c_{2}$ is constrained between −2 and 4. $c_{1}$ and $c_{2}$ are jointly optimized by a two-dimensional grid search, as follows. First, a 11 × 11 grid of values is tried on the admissible range of $c_{1}$ and $c_{2}$. Then, the pair with the lowest score is used as the center of a new 11 × 11 grid, whose total size covers that of four cells of the previous grid. The same procedure yields a refined estimate of $c_{1}$ and $c_{2}$. This pair is in turn used in a second round of refinement, for which another 11 × 11 grid is generated with half the gridsize of the previous round, yielding the final estimate of $c_{1}$ and $c_{2}$. Importantly, before each evaluation of the score at a given $c_{1}$ and $c_{2}$ pair, *σ* and *γ* are set to their maximum posterior estimates.

### 4.3 Rigid Body Self Organizing Map

A 50 × 50 SOM Bouvier et al. (2015), Spill et al. (2013) was trained on the last 200 frames of each of the 64 simulations. Specifically, we used descriptors with seven dimensions, extracted from the structures as follows. The coordinates of all 12,800 structures were recalculated in a reference frame in which the center of mass of PTP is at the origin, and its orientation is constant across the structures. The first three dimensions of the descriptors are the center of mass of PDZ in this reference frame, while the last four are the quaternions of the rotation of PDZ with respect to PTP. The metric used to compare a neuron *n* and a descriptor *m* is a weighted sum between euclidean distance between the center of masses and geodesic distance between the quaternions Huynh (2009).

$$d\left(n,m\right)=\sqrt{\overset{3}{\underset{i=1}{\sum }}{\left(n_{i}-m_{i}\right)}^{2}}+\frac{2d_{\text{max}}}{\pi }\text{arccos}|\overset{7}{\underset{i=4}{\sum }}n_{i}m_{i}|$$ (4)

where $d_{\text{max}}$ is the length of the largest space diagonal of the bounding box of the descriptor’s first three coordinates. Neurons were updated by interpolation either in Cartesian space (first three coordinates) or in quaternion space, e.g., on the unit 4-sphere (last four coordinates).

### 4.4 Linker Modeling

In the second stage, we added linkers to our models. Due to the particular choice of the format of the SOM descriptors, a 3D structure can be reconstructed from the coordinates of the trained neurons. 1,999 clash-free structures could be extracted from the SOM neurons in such a way.

Missing residues were added with IMP Russel et al. (2012) so that the modeled part of the protein spanned residues 496–926. The termini were assigned random ϕ/ψ dihedral angles in such a way that no clash was caused.

The linker was generated in two steps. First, ${\text{C}}_{\alpha }$ atoms were placed on a path that connects the two endpoints without passing through either PTP or PDZ. The ${\text{C}}_{\alpha }$ linker was then minimized with a harmonic distance restraint between consecutive ${\text{C}}_{\alpha }$ atoms (target distance D=3.86Å) and an excluded volume restraint to avoid interpenetration. ${\text{C}}_{\alpha }$ atoms within the linker had a normal diameter *D* while other atoms had diameter 2D to push the linker outside of the protein during initial minimization. 1,000 steps of steepest descent were followed by 1,000 steps of conjugate gradient.

Second, all atoms were placed around their corresponding ${\text{C}}_{\alpha }$ at random in a sphere of diameter *D*. CHARMM bonded restraints were enforced MacKerell et al. (1998), and 250 steps of steepest descent were performed, followed by 1,000 steps of conjugate gradient. Then, volume exclusion was turned on, with standard CHARMM radii, and followed by the same 250 + 1,000 steps of minimization.

On average, this step resulted in 1,224 structures per pose, or a total of 2,461,844 structures.

### 4.5 Monte Carlo Refinement

For each of the 1,999 rigid body poses, the structure with linkers which had the best Bayesian SAXS score was used as starting conformation for a Monte Carlo refinement simulation. Each simulation consisted of 2,000 steps, each of which was an alternation between 10 Monte Carlo moves and optimization of *σ* and *γ*. Each Monte Carlo move was made in internal coordinates, and consisted in a Gaussian perturbation of the backbone dihedrals of residues 496–511, 606–636, and 914–926. The standard deviation of the Gaussian was $5\times 10^{-2}\text{r}ad$ for the termini and $5\times 10^{-3}\text{r}ad$ for the linker.

### 4.6 Fixed-Weight Small-angle X-ray scattering Simulations

To compare fixed-weight and self-adjusting simulations, we used a similar setup. 20 fixed-weight simulations were performed with a SAXS restraint with a weight spaced logarithmically from $10^{-4}$ to $10^{2}$. 10 simulations using the Bayesian SAXS score described here were performed for comparison. The starting structure was always identical, and consisted of a random orientation of PDZ with respect to PTP, with linkers and termini added. Each simulation was performed for 5,000 steps.

### 4.7 Molecular Dynamics Simulations

**FIGURE 11:**
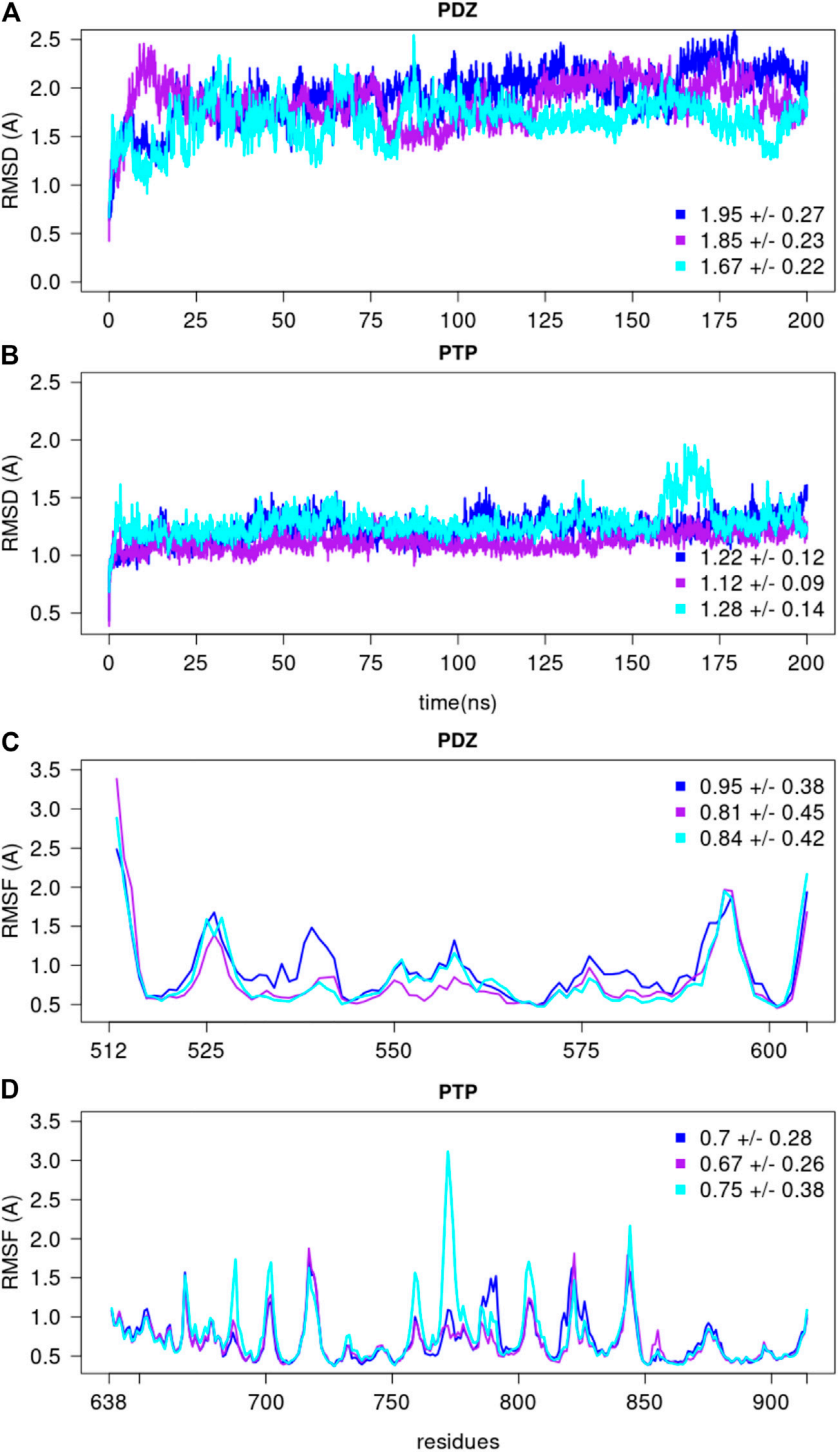
The root mean square deviations and fluctuations of each MD simulation. The RMSD over backbone atoms (C*α*, C, N, O) measured from the initial structure are shown for the **(A)** PDZ and **(B)** PTP domains. The residue RMSF over backbone atoms (C*α*, C, N, O) measured with respect to the average conformation are depicted for the **(C)** PDZ and **(D)** PTP domains, over the last 150 ns of each replicate. The average and standard deviation values are reported for every replicate.

We selected the top PTPN4 conformation determined using the Bayesian SAXS score, i.e., the one with the lowest $\chi^{2}$ score (2.48). This conformation was used as the starting structure for the molecular dynamics simulations (7). MD simulations were performed with NAMD2.13 Phillips et al. (2005) using CHARMM36m force field parameter set Huang et al. (2017): i) hydrogen atoms were added, ii) the solute was hydrated with a cuboid box of explicit TIP3P water molecules Jorgensen et al. (1983) with a buffering distance up to 10 Å, iii) 10 Na+counter-ions were added to neutralise the system, leading to a total system size of 150,730 atoms. The following minimization procedure was applied: i) 10,000 steps of minimization of the water molecules keeping protein atoms fixed, ii) 10,000 steps of minimization keeping only protein backbone fixed to allow protein side chains to relax, iii) 10,000 steps of minimization without any constraint on the system. Heating of the system to the target temperature of 310 K was performed at constant volume using the Berendsen thermostat Berendsen et al. (1984). Thereafter, the system was equilibrated for 100 ps at constant volume (NVT) and for further 100 ps using a Langevin piston (NPT) Loncharich et al. (1992) to maintain the pressure. The production was realised in the NPT ensemble. The time step was set to 2.0 fs. The temperature was kept at 310 K and pressure at 1 bar using the Langevin piston coupling algorithm. The SHAKE algorithm was used to freeze bonds involving hydrogen atoms, allowing for an integration time step of 2.0 fs. The Particle Mesh Ewald method Darden et al. (1993) was employed to treat long-range electrostatics. The coordinates of the system were written every 10 ps. We performed three replicates of 200 ns, with different initial velocities. To assess the stability of each replicate, the root mean square deviation (RMSD) and fluctuation (RMSF) were recorded along each MD simulation (Figure 11). We also measured the distances along the simulations between the center of mass of the two domains in each replicate (Figure 8).

### 4.8 Back Calculated Small-angle X-ray scattering Profiles

For every conformation of the MD simulations, the theoretical scattering profiles were calculated using CRYSOL from the ATSAS 2.8.3 software suite Svergun et al. (1995), with the default parameters. Their corresponding $\chi^{2}$ values were measured using the following equation:

$$\chi^{2}=\frac{1}{M}\overset{M}{\underset{i=1}{\sum }}{\left(\frac{I_{calc}\left(i\right)-I_{exp}\left(i\right)}{\sigma_{exp}\left(i\right)}\right)}^{2}$$ (5)

where *M* is the number of points in SAXS profile, $I_{calc}$ is the back calculated intensity, $I_{exp}$ and $\sigma_{exp}$ are the experimental intensity and error, respectively.

### 4.9 Genetic Algorithm

We followed a similar procedure as in Delhommel et al. (2017), in which 1,000 steps of GA were performed, the number of generated ensemble was set to 1,000 with a cross over frequency of 0.8 and a mutation frequency of one. We performed the GA for different ensemble sizes: 1, 3, 5, 30, and 100. In addition, the GA was repeated five times for every ensemble size and average values were reported.

## Data Availability

The datasets presented in this study can be found in online repositories. The names of the repository/repositories and accession number(s) can be found below: The SAXS data, refined structures, and MD simulation trajectories generated for the PTPN4 for this study are deposited in the Zenodo. org database (accession doi: 10.5281/zenodo.4739101). Direct link: https://zenodo.org/record/4739101.

## References

[B1] Andersen J. N. Mortensen O. H. Peters G. H. Drake P. G. Iversen L. F. Olsen O. H. (2001). Structural and Evolutionary Relationships Among Protein Tyrosine Phosphatase Domains. Mol. Cel. Biol. 21, 7117–7136. 10.1128/mcb.21.21.7117-7136.2001 PMC99888 11585896

[B2] Babault N. Cordier F. Lafage M. Cockburn J. Haouz A. Prehaud C. (2011). Peptides Targeting the PDZ Domain of PTPN4 Are Efficient Inducers of Glioblastoma Cell Death. Structure 19, 1518–1524. 10.1016/j.str.2011.07.007 22000519

[B3] Barr A. J. Ugochukwu E. Lee W. H. King O. N. F. Filippakopoulos P. Alfano I. (2009). Large-scale Structural Analysis of the Classical Human Protein Tyrosine Phosphatome. Cell 136, 352–363. 10.1016/j.cell.2008.11.038 19167335 PMC2638020

[B4] Berendsen H. J. C. Postma J. P. M. van Gunsteren W. F. DiNola A. Haak J. R. (1984). Molecular Dynamics with Coupling to an External bath. J. Chem. Phys. 81, 3684–3690. 10.1063/1.448118

[B5] Bernard A. Vranken W. F. Bardiaux B. Nilges M. Malliavin T. E. (2011). Bayesian Estimation of NMR Restraint Potential and Weight: a Validation on a Representative Set of Protein Structures. Proteins 79, 1525–1537. 10.1002/prot.22980 21365680

[B6] Bonomi M. Hanot S. Greenberg C. H. Sali A. Nilges M. Vendruscolo M. (2019). Bayesian Weighing of Electron Cryo-Microscopy Data for Integrative Structural Modeling. Structure 27, 175–188. 10.1016/j.str.2018.09.011 30393052 PMC6779587

[B7] Bouvier G. Desdouits N. Ferber M. Blondel A. Nilges M. (2015). An Automatic Tool to Analyze and Cluster Macromolecular Conformations Based on Self-Organizing Maps. Bioinformatics 31, 1490–1492. 10.1093/bioinformatics/btu849 25543048

[B8] Brünger A. T. (1992). Free R Value: a Novel Statistical Quantity for Assessing the Accuracy of crystal Structures. Nature 355, 472–475. 10.1038/355472a0 18481394

[B9] Caillet-Saguy C. Maisonneuve P. Delhommel F. Terrien E. Babault N. Lafon M. (2015). Strategies to Interfere with PDZ-Mediated Interactions in Neurons: What We Can Learn from the Rabies Virus. Prog. Biophys. Mol. Biol. 119, 53–59. 10.1016/j.pbiomolbio.2015.02.007 25748547

[B10] Caillet-Saguy C. Toto A. Guerois R. Maisonneuve P. Di Silvio E. Sawyer K. (2017). Regulation of the Human Phosphatase PTPN4 by the Interdomain Linker Connecting the PDZ and the Phosphatase Domains. Scientific Rep. 7, 2–10. 10.1038/s41598-017-08193-6 PMC5554198 28801650

[B11] Chen P.-c. Hub J. S. (2015). Interpretation of Solution X-ray Scattering by Explicit-Solvent Molecular Dynamics. Biophysical J. 108, 2573–2584. 10.1016/j.bpj.2015.03.062 PMC4457003 25992735

[B12] Darden T. York D. Pedersen L. (1993). Particle Mesh Ewald: AnN⋅Log(N) Method for Ewald Sums in Large Systems. J. Chem. Phys. 98, 10089–10092. 10.1063/1.464397

[B13] Delhommel F. Cordier F. Bardiaux B. Bouvier G. Colcombet-Cazenave B. Brier S. (2017). Structural Characterization of Whirlin Reveals an Unexpected and Dynamic Supramodule Conformation of its PDZ Tandem. Structure 25, 1645–1656. 10.1016/j.str.2017.08.013 28966015

[B14] Dill K. A. Chan H. S. (1997). From Levinthal to Pathways to Funnels: The ”New View” of Protein Folding Kinetics. Nat. Struct. Biol. 4, 10. 8989315 10.1038/nsb0197-10

[B15] Ferber M. Kosinski J. Ori A. Rashid U. J. Moreno-Morcillo M. Simon B. (2016). Automated Structure Modeling of Large Protein Assemblies Using Crosslinks as Distance Restraints. Nat. Methods 13, 515–520. 10.1038/nmeth.3838 27111507

[B16] Gu M. Majerus P. W. (1996). The Properties of the Protein Tyrosine Phosphatase PTPMEG. J. Biol. Chem. 271, 27751–27759. 10.1074/jbc.271.44.27751 8910369

[B17] Gu M. Meng K. Majerus P. W. (1996). The Effect of Overexpression of the Protein Tyrosine Phosphatase PTPMEG on Cell Growth and on colony Formation in Soft agar in COS-7 Cells. Proc. Natl. Acad. Sci. 93, 12980–12985. 10.1073/pnas.93.23.12980 8917530 PMC24032

[B18] Habeck M. Rieping W. Nilges M. (2006). Weighting of Experimental Evidence in Macromolecular Structure Determination. Proc. Natl. Acad. Sci. 103, 1756–1761. 10.1073/pnas.0506412103 16446450 PMC1413624

[B19] Huang J. Rauscher S. Nawrocki G. Ran T. Feig M. de Groot B. L. (2017). CHARMM36m: an Improved Force Field for Folded and Intrinsically Disordered Proteins. Nat. Methods 14, 71–73. 10.1038/nmeth.4067 27819658 PMC5199616

[B20] Huynh D. Q. (2009). Metrics for 3d Rotations: Comparison and Analysis. J. Math. Imaging Vis. 35, 155–164. 10.1007/s10851-009-0161-2

[B21] Jorgensen W. L. Chandrasekhar J. Madura J. D. Impey R. W. Klein M. L. (1983). Comparison of Simple Potential Functions for Simulating Liquid Water. J. Chem. Phys. 79, 926–935. 10.1063/1.445869

[B22] Kina S.-i. Tezuka T. Kusakawa S. Kishimoto Y. Kakizawa S. Hashimoto K. (2007). Involvement of Protein-Tyrosine Phosphatase PTPMEG in Motor Learning and Cerebellar Long-Term Depression. Eur. J. Neurosci. 26, 2269–2278. 10.1111/j.1460-9568.2007.05829.x 17953619

[B23] Kohda K. Kakegawa W. Matsuda S. Yamamoto T. Hirano H. Yuzaki M. (2013). The 2 Glutamate Receptor gates Long-Term Depression by Coordinating Interactions between Two AMPA Receptor Phosphorylation Sites. Proc. Natl. Acad. Sci. 110, E948–E957. 10.1073/pnas.1218380110 23431139 PMC3593918

[B24] Loncharich R. J. Brooks B. R. Pastor R. W. (1992). Langevin Dynamics of Peptides: The Frictional Dependence of Isomerization Rates ofN-Acetylalanyl-N?-Methylamide. Biopolymers 32, 523–535. 10.1002/bip.360320508 1515543

[B25] MacKerell A. D. Bashford D. Bellott M. Dunbrack R. L. Evanseck J. D. Field M. J. (1998). All-Atom Empirical Potential for Molecular Modeling and Dynamics Studies of Proteins†. J. Phys. Chem. B 102, 3586–3616. 10.1021/jp973084f 24889800

[B26] Maisonneuve P. Caillet-Saguy C. Raynal B. Gilquin B. Chaffotte A. Pérez J. (2014). Regulation of the Catalytic Activity of the Human Phosphatase Ptpn4 by its Pdz Domain. Febs J. 281, 4852–4865. 10.1111/febs.13024 25158884

[B27] Maisonneuve P. Caillet-Saguy C. Vaney M.-C. Bibi-Zainab E. Sawyer K. Raynal B. (2016). Molecular Basis of the Interaction of the Human Protein Tyrosine Phosphatase Non-receptor Type 4 (PTPN4) with the Mitogen-Activated Protein Kinase P38γ. J. Biol. Chem. 291, 16699–16708. 10.1074/jbc.m115.707208 27246854 PMC4974383

[B28] Mareuil F. Sizun C. Perez J. Schoenauer M. Lallemand J.-Y. Bontems F. (2007). A Simple Genetic Algorithm for the Optimization of Multidomain Protein Homology Models Driven by NMR Residual Dipolar Coupling and Small Angle X-ray Scattering Data. Eur. Biophys. J. 37, 95–104. 10.1007/s00249-007-0170-2 17522855

[B29] Nilges M. Bernard A. Bardiaux B. Malliavin T. Habeck M. Rieping W. (2008). Accurate NMR Structures through Minimization of an Extended Hybrid Energy. Structure 16, 1305–1312. 10.1016/j.str.2008.07.008 18786394

[B30] Paissoni C. Jussupow A. Camilloni C. (2020). Determination of Protein Structural Ensembles by Hybrid-Resolution SAXS Restrained Molecular Dynamics. J. Chem. Theor. Comput. 16, 2825–2834. 10.1021/acs.jctc.9b01181 PMC7997378 32119546

[B31] Phillips J. C. Braun R. Wang W. Gumbart J. Tajkhorshid E. Villa E. (2005). Scalable Molecular Dynamics with NAMD. J. Comput. Chem. 26, 1781–1802. 10.1002/jcc.20289 16222654 PMC2486339

[B32] Potrzebowski W. Trewhella J. Andre I. (2018). Bayesian Inference of Protein Conformational Ensembles from Limited Structural Data. Plos Comput. Biol. 14, e1006641. 10.1371/journal.pcbi.1006641 30557358 PMC6312354

[B33] Préhaud C. Wolff N. Terrien E. Lafage M. Mégret F. Babault N. (2010). Attenuation of Rabies Virulence: Takeover by the Cytoplasmic Domain of its Envelope Protein. Sci. Signaling 3, ra5. 10.1126/scisignal.2000510 20086240

[B34] Rieping W. Habeck M. Nilges M. (2005). Inferential Structure Determination. Science 309, 303–306. 10.1126/science.1110428 16002620

[B35] Rozycki B. Kim Y. C. Hummer G. (2011). SAXS Ensemble Refinement of ESCRT-III CHMP3 Conformational Transitions. Structure 19, 109–116. 21220121 10.1016/j.str.2010.10.006 PMC3032427

[B36] Russel D. Lasker K. Webb B. Velázquez-Muriel J. Tjioe E. Schneidman-Duhovny D. (2012). Putting the Pieces Together: Integrative Modeling Platform Software for Structure Determination of Macromolecular Assemblies. Plos Biol. 10, e1001244. 10.1371/journal.pbio.1001244 22272186 PMC3260315

[B37] Schneidman-Duhovny D. Hammel M. Tainer J. A. Sali A. (2013). Accurate SAXS Profile Computation and its Assessment by Contrast Variation Experiments. Biophysical J. 105, 962–974. 10.1016/j.bpj.2013.07.020 PMC3752106 23972848

[B38] Shevchuk R. Hub J. S. (2017). Bayesian Refinement of Protein Structures and Ensembles against SAXS Data Using Molecular Dynamics. Plos Comput. Biol. 13, e1005800. 10.1371/journal.pcbi.1005800 29045407 PMC5662244

[B39] Shrestha U. R. Juneja P. Zhang Q. Gurumoorthy V. Borreguero J. M. Urban V. (2019). Generation of the Configurational Ensemble of an Intrinsically Disordered Protein from Unbiased Molecular Dynamics Simulation. Proc. Natl. Acad. Sci. USA 116, 20446–20452. 10.1073/pnas.1907251116 31548393 PMC6789927

[B40] Spill Y. (2013). Développement de méthodes d’échantillonnage et traitement bayésien de données continues: nouvelle méthode d’échange de répliques et modélisation de données SAXS. Ph.D. Thesis, Paris 7.

[B41] Spill Y. G. Bouvier G. Nilges M. (2013). A Convective Replica-Exchange Method for Sampling New Energy Basins. J. Comput. Chem. 34, 132–140. 10.1002/jcc.23113 22961200

[B42] Spill Y. G. Kim S. J. Schneidman-Duhovny D. Russel D. Webb B. Sali A. (2014). Saxs Merge: an Automated Statistical Method to Merge Saxs Profiles Using Gaussian Processes. J. Synchrotron Radiat. 21, 203–208. 10.1107/s1600577513030117 24365937 PMC3874021

[B43] Svergun D. Barberato C. Koch M. H. J. (1995). CRYSOL- a Program to Evaluate X-ray Solution Scattering of Biological Macromolecules from Atomic Coordinates. J. Appl. Cryst. 28, 768–773. 10.1107/s0021889895007047

[B44] Yang S. Blachowicz L. Makowski L. Roux B. (2010). Multidomain Assembled States of Hck Tyrosine Kinase in Solution. Proc. Natl. Acad. Sci. 107, 15757–15762. 10.1073/pnas.1004569107 20798061 PMC2936629

[B45] Young J. A. Becker A. M. Medeiros J. J. Shapiro V. S. Wang A. Farrar J. D. (2008). The Protein Tyrosine Phosphatase PTPN4/PTP-MEG1, an Enzyme Capable of Dephosphorylating the TCR ITAMs and Regulating NF-Κb, Is Dispensable for T Cell Development And/or T Cell Effector Functions. Mol. Immunol. 45, 3756–3766. 10.1016/j.molimm.2008.05.023 18614237 PMC2596642

[B46] Zhang B. D. Li Y. R. Ding L. D. Wang Y. Y. Liu H. Y. Jia B. Q. (2019). Loss of PTPN4 Activates STAT3 to Promote the Tumor Growth in Rectal Cancer. Cancer Sci. 110, 2258–2272. 10.1111/cas.14031 31025789 PMC6609803

[B47] Zhou J. Wan B. Shan J. Shi H. Li Y. Huo K. (2013). PTPN4 Negatively Regulates CrkI in Human Cell Lines. Cell Mol Biol Lett 18, 297–314. 10.2478/s11658-013-0090-3 23666597 PMC6275623

