# Supplementary material for: Automatic Bayesian Weighting for SAXS Data
Source: Front Mol Biosci. 2021 Jun 4;8:671011. doi: 10.3389/fmolb.2021.671011 (PMC8212126; doi:10.3389/fmolb.2021.671011)
Supplement: Supplementary file 1 [file DataSheet1.PDF]

frontiers

Supplementary Material

1 DERIVATION OF THE BAYESIAN SAXS RESTRAINT

Define

$$\mathbf{I} = \begin{pmatrix} I(q_1) \\ \vdots \\ I(q_M) \end{pmatrix} \qquad \mathbf{S} = \mathrm{diag}\begin{pmatrix} s(q_1)^2 \\ \vdots \\ s(q_M)^2 \end{pmatrix} \qquad \mathcal{I} = \begin{pmatrix} \mathcal{I}(q_1) \\ \vdots \\ \mathcal{I}(q_M) \end{pmatrix}$$

$\mathbf{I}$ is a vector of observed SAXS intensities of length M, $\mathbf{S}$ is the diagonal matrix of sample variances, and $\mathcal{I}$ is the unknown SAXS profile which was observed with gaussian noise and up to a scaling factor: $\mathbf{I} \sim \mathcal{N}(\gamma\mathcal{I}, \sigma^2\mathbf{S})$. Here, $\mathbf{S}$ is assumed to be diagonal, but the derivation is not affected by this fact. If available, covariances, such as those measured in Franke et al. (2015), could also be included in $\mathbf{S}$. Suppose furthermore that X is the structure to be determined. The likelihood is then defined as the marginal distribution

$$p(\mathbf{I}|X, \gamma, \sigma^2) = \int \mathrm{d}\mathcal{I} p(\mathbf{I}|\mathcal{I}, \gamma, \sigma^2) p(\mathcal{I}|X)$$

Conditional on $\mathcal{I}$, data points are independent of each other. The distribution of $\mathbf{I}$ given $\mathcal{I}$ is therefore a product of standard normal distributions around the true mean of each SAXS profile

$$p(\mathbf{I}|\mathcal{I}, \gamma, \sigma^2) = \prod_{i=1}^{M} \frac{1}{\sqrt{2\pi}\sigma s(q_i)} \exp\left(-\frac{1}{2}\left(\frac{I(q_i) - \gamma\mathcal{I}(q_i)}{\sigma s(q_i)}\right)^2\right) \tag{S1}$$

$\mathcal{I}$ is the calculated scattering intensity, as given by a number of forward models based on X. Such models can be based on a single atomic or pseudo-atomic structure Svergun et al. (1995); Schneidman-Duhovny et al. (2013); Poitevin et al. (2011), based on an MD simulation Merzel and Smith (2002); Park et al. (2009); Köfinger and Hummer (2013); Chen and Hub (2014), based on a discrete ensemble of structures Bernadó et al. (2007); Pelikan M. (2009); Yang et al. (2010); Rozycki et al. (2011); Berlin et al. (2013) or any other method, depending on how X is defined. In that case, $\mathcal{I} = m(X)$ where m is the forward model, and $p(\mathbf{I}|X, \gamma, \sigma^2)$ is given by eq. S1. The minus log likelihood is therefore, up to a constant term

$$-\log p(\mathbf{I}|X, \gamma, \sigma^2) = \frac{M}{2\sigma^2}\chi^2 + M\log(\sigma) \tag{S2}$$

$$\chi^2 \equiv \frac{1}{M}\sum_{i=1}^{M}\left(\frac{I(q_i) - \gamma m(X, q_i)}{s(q_i)}\right)^2 \tag{S3}$$

Using an uninformative prior such as $p(\sigma^2) \propto 1/\sigma^2$, we can obtain the optimal value for σ^2 at fixed χ^2 in closed form, and is

$$\sigma^2 = \frac{M}{M+2}\chi^2 \tag{S4}$$

Further, we can marginalize σ using this prior and obtain

$$p(\mathbf{I}|X,\gamma) = \int_0^{+\infty} \mathrm{d}\sigma^2 p(\mathbf{I}|X,\sigma^2,\gamma)p(\sigma^2) = \frac{\Gamma(\frac{M}{2})}{2(2\pi)^{\frac{M}{2}}}\,\chi^{-M}$$

(with $\chi \equiv \sqrt{\chi^2}$). Therefore, up to an additive constant,

$$-\log p(\mathbf{I}|X,\gamma) = M\log\chi \tag{S5}$$

To extend this model, it is also possible to relax the link between $\mathcal{I}$ and the forward model $m(X)$. A tractable model is when $\mathcal{I}$ is drawn from a multivariate normal distribution with mean vector $\mathbf{m}$ and covariance matrix $\mathbf{\Sigma}$.

$$p(\mathcal{I}|X) = \frac{1}{(2\pi)^{\frac{M}{2}}|\Sigma|^{1/2}}\exp\left(-\frac{1}{2}(\mathcal{I}-\mathbf{m})^\top\mathbf{\Sigma}^{-1}(\mathcal{I}-\mathbf{m})\right)$$

The construction of $\mathbf{m}$ and $\mathbf{\Sigma}$ is described in detail in Spill and Nilges (2017). Because likelihood and prior are both normal distributions, we can marginalize $\mathcal{I}$, obtaining

$$p(\mathbf{I}|X,\gamma,\sigma^2) = \frac{1}{(2\pi)^{\frac{M}{2}}|\mathbf{\Omega}|^{1/2}}\exp\left(-\frac{1}{2}\varepsilon^\top\mathbf{\Omega}^{-1}\varepsilon\right) \tag{S6}$$

$$\varepsilon \equiv \mathbf{I}-\gamma\mathbf{m} \qquad \mathbf{\Omega} = \sigma^2\mathbf{S}+\gamma^2\mathbf{\Sigma}$$

The minus log likelihood is therefore, up to a constant term

$$-\log p(\mathbf{I}|X,\gamma,\sigma^2) = \frac{M}{2\sigma^2}\chi^2 + \frac{1}{2}\log|\mathbf{\Omega}| \tag{S7}$$

$$\chi^2 \equiv \frac{\sigma^2}{M}\varepsilon^\top\mathbf{\Omega}^{-1}\varepsilon \tag{S8}$$

Note that equation S7 (resp. S8) reverts to equation S2 (resp. S3) when $\mathbf{\Sigma} = 0$. Also, σ cannot be marginalized in this case, that is, eq. S5 cannot be generalized.

REFERENCES

Berlin, K., Castañeda, C. A., Schneidman-Duhovny, D., Sali, A., Nava-Tudela, A., and Fushman, D. (2013). Recovering a representative conformational ensemble from underdetermined macromolecular structural data. *J. Am. Chem. Soc.* 135, 16595–16609. doi:10.1021/ja4083717

Bernadó, P., Mylonas, E., Petoukhov, M. V., Blackledge, M., and Svergun, D. I. (2007). Structural characterization of flexible proteins using small-angle x-ray scattering. *J. Am. Chem. Soc.* 129, 5656–5664. doi:10.1021/ja069124n

Chen, P. and Hub, J. S. (2014). Validating solution ensembles from molecular dynamics simulation by wide-angle x-ray scattering data. *Biophys. J.* 107, 435 – 447. doi:10.1016/j.bpj.2014.06.006

Franke, D., Jeffries, C. M., and Svergun, D. I. (2015). Correlation map, a goodness-of-fit test for one-dimensional x-ray scattering spectra. *Nat. Methods* 12, 419–422. doi:10.1038/nmeth.3358

Köfinger, J. and Hummer, G. (2013). Atomic-resolution structural information from scattering experiments on macromolecules in solution. *Phys. Rev. E* 87, 052712. doi:10.1103/PhysRevE.87.052712

Merzel, F. and Smith, J. C. (2002). Is the first hydration shell of lysozyme of higher density than bulk water? *Proc. Nat. Acad. Sci. USA* 99, 5378–5383. doi:10.1073/pnas.082335099

Park, S., Bardhan, J. P., Roux, B., and Makowski, L. (2009). Simulated x-ray scattering of protein solutions using explicit-solvent models. *J. Chem. Phys.* 130, 134114–134118. doi:10.1063/1.3099611

Pelikan M., H. M., Hura G. L. (2009). Structure and flexibility within proteins as identified through small angle x-ray scattering. *Gen. Physiol. Biophys.* 28, 174–189. doi:10.4149/gpb-2009-02-174

Poitevin, F., Orland, H., Doniach, S., Koehl, P., and Delarue, M. (2011). AquaSAXS: a web server for computation and fitting of SAXS profiles with non-uniformally hydrated atomic models. *Nucleic Acids Research* 39, W184–W189. doi:10.1093/nar/gkr430

Rozycki, B., Kim, Y. C., and Hummer, G. (2011). SAXS ensemble refinement of ESCRT-III CHMP3 conformational transitions. *Structure* 19, 109 – 116. doi:10.1016/j.str.2010.10.006

Schneidman-Duhovny, D., Hammel, M., Tainer, J. A., and Sali, A. (2013). Accurate SAXS profile computation and its assessment by contrast variation experiments. *Biophys. J.* 105, 962 – 974. doi:10.1016/j.bpj.2013.07.020

Spill, Y. G. and Nilges, M. (2017). Sas profile correlations reveal sas hierarchical nature and information content. *PLoS One* 12, e0177309. doi:10.1371/journal.pone.0177309

Svergun, D. I., Barberato, C., and Koch, M. H. J. (1995). CRYSOL – a Program to Evaluate X-ray Solution Scattering of Biological Macromolecules from Atomic Coordinates. *Journal of Applied Crystallography* 28, 768–773. doi:10.1107/S0021889895007047

Yang, S., Blachowicz, L., Makowski, L., and Roux, B. (2010). Multidomain assembled states of hck tyrosine kinase in solution. *Proc. Nat. Acad. Sci. USA* 107, 15757–15762. doi:10.1073/pnas.1004569107
